# Time-Resolved ATR–FTIR Spectroscopy and Macro ATR–FTIR Spectroscopic Imaging of Inorganic Treatments for Stone Conservation

**DOI:** 10.1021/acs.analchem.1c02392

**Published:** 2021-10-26

**Authors:** Elena Possenti, Chiara Colombo, Marco Realini, Cai Li Song, Sergei G. Kazarian

**Affiliations:** † Istituto di Scienze del Patrimonio Culturale, Consiglio Nazionale delle Ricerche, ISPC-CNR, Via R. Cozzi 53, Milano 20125, Italy; ‡ Department of Chemical Engineering, Imperial College London, South Kensington Campus London, London SW7 2AZ, United Kingdom

## Abstract

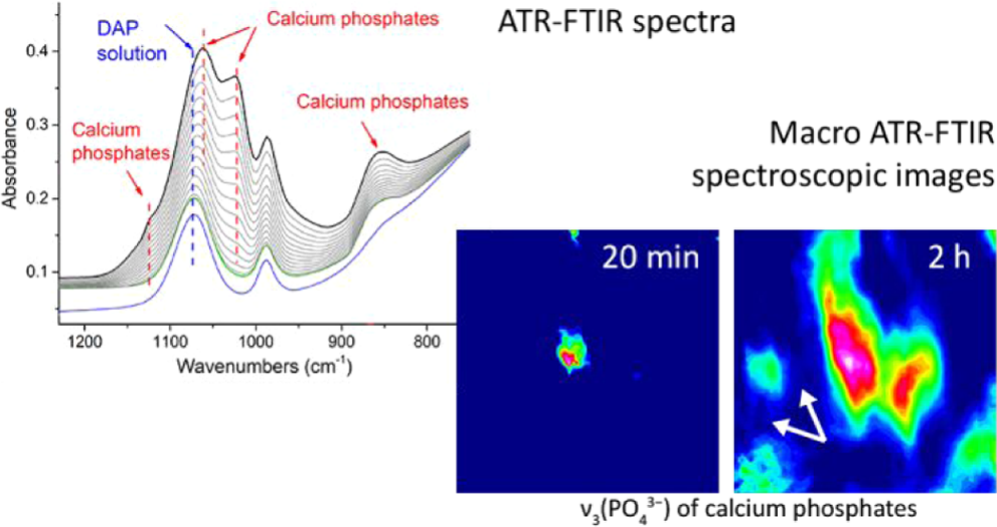

In this study, the novel application of ATR–FTIR spectroscopy and macro ATR–FTIR spectroscopic imaging overcame an analytical challenge in conservation science: the time-resolved, chemical, and spatial investigation of the reaction of inorganic treatments for stone conservation (ammonium oxalate, AmOx; ammonium phosphate, DAP) occurring in water-based solutions. The aim was to (1) assess the composition and localization of reaction products and their phase variation during the reaction in real time and directly in an aqueous environment and (2) investigate the reaction of AmOx and DAP with calcite and the transformations induced to the substrate with a time-resolved approach. The new analytical results showed that for both treatments, the formation of new crystalline phases initiated at the early stages of the reaction. Their composition changed during the treatment and led to more stable phases. The reactivity of the stone substrate to the treatments varied as a function of the stone material features, such as the specific surface area. A clear influence of post-treatment rinsing on the final composition of reaction phases was observed. Above all, our research demonstrates the actual feasibility, practicality, and high potential of an advanced ATR–FTIR spectroscopic approach to investigate the behavior of conservation treatments and provided new analytical tools to address the choices of conservation in pilot worksites. Lastly, this study opens novel analytical perspectives based on the new possible applications of ATR–FTIR spectroscopic imaging in the field of conservation science, materials science, and analytical chemistry.

## Introduction

Attenuated total reflection–Fourier transform infrared (ATR–FTIR) spectroscopy is a powerful and versatile analytical technique that provides qualitative and quantitative information with high chemical specificity. ATR–FTIR spectroscopy has long been applied in industry and research to characterize a wide range of materials, even in complex mixtures. In addition, ATR–FTIR spectroscopy allows us to investigate the kinetics of chemical reactions occurring in solutions, including those in aqueous environments. This makes ATR–FTIR spectroscopy a potential approach to provide important information in case of analytical challenges, such as the time-resolved investigations of chemical reactions occurring in a water-based solution when inorganic mineral treatments are applied.

Two inorganic mineral treatments are commonly used for the conservation of carbonatic stone materials: ammonium oxalate [AmOx, (NH_4_)_2_C_2_O_4_·H_2_O) and diammonium hydrogen phosphate (DAP, (NH_4_)_2_HPO_4_].^1^ Both the treatments are based on water soluble products (AmOx and DAP) that react with calcite of the substrate to form calcium oxalates and calcium phosphates through a dissolution–recrystallization process.^1,2^ The reaction occurs at the interface between the grains of the stone material and the water-based solution of the treatment, and it promotes the crystallization of newly formed phases arranged in a micrometer-size shell on the reaction profile of calcite grains.^3^ The shell of the new phases provides a consolidating and/or protective action by reconnecting detached grains or limiting the interactions with the surrounding environment. The low solubility of the reaction products even to acid environments provides an advantageous acid resistance for carbonatic substrates exposed to polluted urban regions.^1^

By forming new crystalline phases, these conservation treatments irreversibly modify the mineralogical composition, the microstructural features, and the mechanical–physical properties of the original materials.^4^ The nature and extent of these variations depend on several mutually interacting variables. Different crystalline phases are formed depending on the reaction conditions (i.e., pH, reagent molarity, ionic strength, mineralogical composition of the substrate, etc).^5−7^ This is a crucial step, as each crystalline phase has specific properties (stability and solubility), and many of them are metastable or/and hydrated phases.^2,8^ No less importantly, the presence of water molecules is crucial for the dissolution–recrystallization (e.g., the evaporation of the solution interrupts the reaction) and to promote the formation of hydrated phases.

The presence of water influences the composition of some crystalline phases even after the treatment. The transformation of metastable hydrated phases in other phases due to relative humidity has been demonstrated in the case of some calcium phosphates formed after DAP treatments,^3,9^ and of dihydrate–monohydrate calcium oxalates after AmOX ones.^10^ These studies pointed out the importance of investigating the conditions of crystallization and the metastability of some phases. A typical step in conservation science is to decide if and how to rinse the treated materials at the end of inorganic conservation treatments. This step aims to remove any possible unreacted reagent from the stone material, but very little is known about the potential effect of liquid water on the crystallo-chemical composition of the newly formed (potentially metastable) reaction products. In fact, post-treatment washing might promote the formation of stable and well-ordered phases. Furthermore, it is conceivable that phase variations, with the temporary crystallization of metastable phases, may also occur during the reaction of inorganic mineral treatments.

Research studies on AmOx and/or DAP treatments are constantly expanding and gaining more interest nowadays.^11−14^

Several analytical techniques, including X-ray diffraction, Raman spectroscopy, neutron tomography–radiography, and IR spectroscopy (conventional FTIR instruments;^15^ micro ATR-FTIR spectroscopic mapping^3,16^), have been used to investigate the composition of the newly formed reaction products^3,6,7,9^ and their penetration within the substrate.^13,16−20^

However, these studies did not focus on the kinetics of the reaction, and further insights are needed about the real-time variations occurring during the conservation treatments and directly in their reaction environment. In this direction, a clear step forward can be carried out using an advanced ATR–FTIR spectroscopic approach based on conventional ATR–FTIR spectroscopy and ATR–FTIR spectroscopic imaging.

In fact, the use of conventional ATR–FTIR spectroscopy allows for the investigation of the fingerprint region over the whole mid-IR range while the use of ATR–FTIR spectroscopic imaging (both macro and micro) combines a spatially resolved outcome with the molecular characterization of the analytes, providing crucial information on the composition and localization of compounds at the microscale.^21−24^ ATR–FTIR spectroscopic imaging has already been used to study complex and heterogeneous samples in several research fields, including pharmaceuticals,^25,26^ biological systems,^21,27^ forensic and materials science,^22,28−30^ and cultural heritage (paint stratigraphies,^31−37^ historical leather book covers,^38^ and albumen photographic prints^39^). Remarkably, in many of these studies, ATR–FTIR spectroscopic imaging measurements were carried out in the presence of liquid water.^21,25,26,28,29,33^

In this paper, a novel analytical application of ATR–FTIR spectroscopy and macro ATR–FTIR spectroscopic imaging for stone conservation is shown. For the first time, we demonstrate the feasibility, practicality, and potential of ATR–FTIR spectroscopy and macro ATR–FTIR spectroscopic imaging to explore the nucleation and growth of IR-active reaction products formed during inorganic-mineral treatments on carbonatic stone substrates directly in the aqueous environment, in a time-resolved and nondestructive way. In this research, our time-resolved ATR–FTIR spectroscopic approach has been applied to investigate the following: (i) chemical composition and phase variation of reaction products formed during AmOx and DAP treatments; (ii) reactivity of the stone substrate to AmOx as a function of the specific surface; and (iii) growth and spatial distribution of the consolidating shell formed during DAP treatments.

## Experimental Section

### Materials

This study was carried out on the veined variety of white Carrara marble, a compact metamorphic lithotype outcropping in the Colonnata basin (Gioia quarry) and widely used in Italy as an ornamental stone.^40^ The lithotype is mainly composed of calcite, with muscovite, quartz, pyrite, albite, chlorite, and dolomite as trace minerals.

The measurements were carried out on the freshly quarried lithotype both in the form of slabs and powders.^9^ The powders were prepared via an artificial weathering method (modified from Franzoni et al.^41^) to obtain the sugaring, a typical decay of marbles.^9^ The slabs and powders of Carrara marbles were used to simulate the well-preserved and highly decayed stone substrates, respectively.

Ammonium oxalate (AmOx, (NH_4_)_2_C_2_O_4_·H_2_O, CAS number 6009-70-7, assay ≥98.0%, reagent grade) and ammonium phosphate (DAP, (NH_4_)_2_HPO_4_, CAS number 7783-28-0, assay ≥99.0%, reagent grade) were purchased from Sigma-Aldrich. A 5% w/w AmOx aqueous solution (0.35 M) and a 10% w/w DAP aqueous solution (0.76 M) were used for the experiments. The molarity of reagents was selected on the basis of previous experiments and practice in the field.^3,4,7,18^

### Methods

The investigations were carried out simultaneously by conventional ATR–FTIR spectroscopy and macro ATR–FTIR spectroscopic imaging.

The former was carried out using a Czitek SurveyIR infrared microscopy accessory (Specac Ltd., UK) equipped with a prism-shaped diamond ATR crystal inserted in a Tensor 27 spectrometer (Bruker Corp.) mounting a DTGS detector. ATR–FTIR spectra were acquired over the 4000–400 cm^–1^ mid-IR range.

The latter was carried out using an imaging Golden Gate ATR accessory (Specac Ltd., UK) with a prism-shaped diamond ATR crystal hosted in an IMAC macrochamber attached to a Bruker Tensor 27 FTIR spectrometer equipped with a 64 × 64-pixel FPA detector (Santa Barbara Focal plane, USA). ATR–FTIR spectra were collected over the 3900–900 cm^–1^ spectral range, with a spatial resolution approximately of 15 μm in macro imaging mode. This setup provided an imaging area of about 0.6 × 0.55 mm. The chemical images were obtained by plotting the distribution of the integrated absorbance of specific spectral bands. The integrated absorbance is shown in a false color scale where magenta/white stands for highest and blue for lowest absorbance. Macro ATR–FTIR spectroscopic imaging was only carried out on powders, as the slight indentation of the diamond ATR crystal prevented a good contact with the hard and noneasily deformed slabs of Carrara marble.

All the ATR–FTIR spectroscopic measurements were taken at 8 cm^–1^ spectral resolution with 64 co-added scans, which allowed the crystallization and the evolution of phases to be monitored during the reaction in a reasonably short time and with a good signal-to-noise ratio.

The stone sample (slab or powder) was put in contact with the ATR crystal. The first measurement was collected without adding any reagent, and it corresponded to the spectroscopic features of the untreated lithotype. Then, a drop of solution was put close to the ATR crystal by using a pipette and without moving the sample. The solution spread itself at the interface between the calcite grains of the stone substrate and the ATR crystal (condition of optimal contact). This spreading was checked in real time by investigating the presence of the vibrational bands of water. At positive check, the monitoring of the reaction between inorganic mineral treatments and Carrara marble was carried out. The acquisition time of a spectrum was approximately 150 s. The ATR–FTIR spectra were recorded at a time interval of 10 min for 12 or 15 consequential measurements (depending on the specific experimental setup), in line with the treatment duration stated in previous studies.^4,7^ The stone samples were not moved throughout the measurements. At the end of the treatments, the composition of the reaction products was also investigated without the solution: the stone substrate was rinsed twice with deionized water, dried at room temperature, and measured again. This last step was possible only for marble slabs. A scheme of the timeline used for the measurements and of the experimental setup is shown in Figure S1 in the Supporting Information.

The FTIR data sets were collected and analyzed with commercial software OPUS (Bruker Corp). As for the integrated absorbance of the spectral bands in the ATR–FTIR spectra, a straight line has been drawn between selected positions in the band at particular wavenumbers. The area above this line has been integrated. The spectroscopic experiments were done at Imperial College London, UK.

## Results and Discussion

### Ammonium Oxalate (AmOx) Treatment

**Figure 1:**
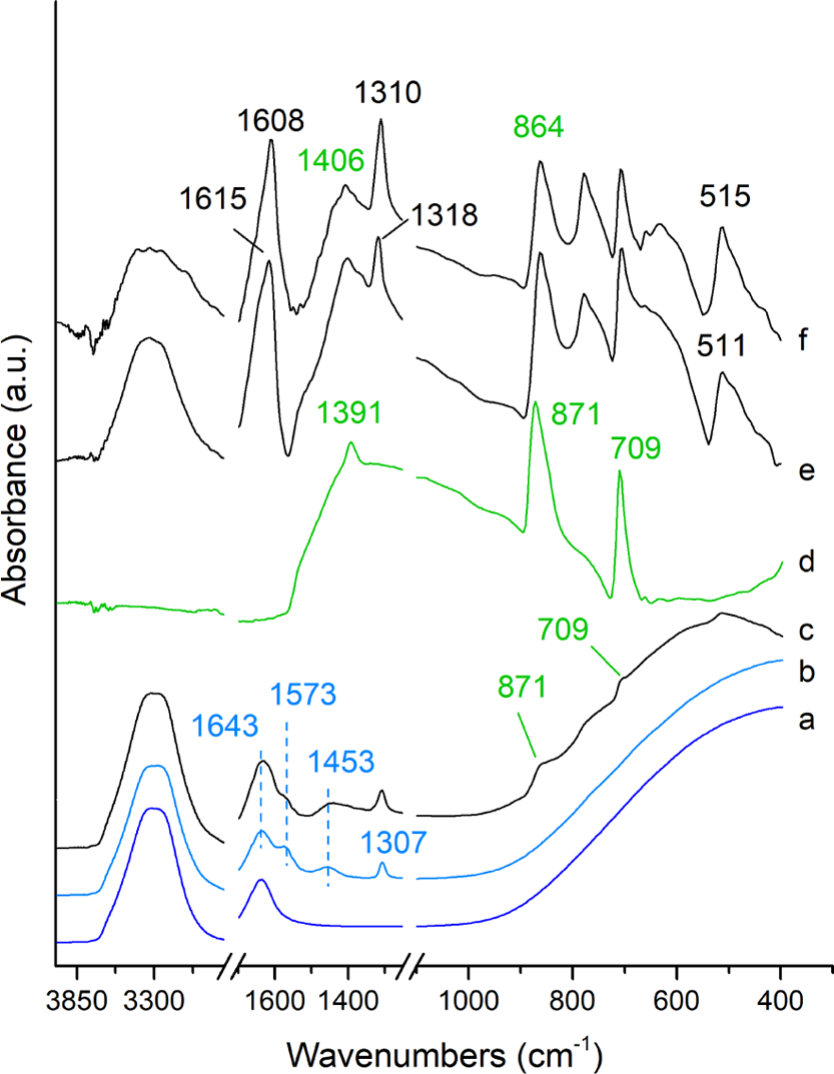
AmOx treatment. ATR–FTIR spectra of the following: deionized water (a), AmOx solution (b), AmOx solution applied to Carrara marble (c), untreated Carrara marble (d), and Carrara marble slab after 2′30″ (e) and 142′30″ (f) from the beginning of the AmOx treatment. The ATR–FTIR spectra (e,f) are obtained by spectral subtraction of the AmOx solution.

The ATR–FTIR spectra of AmOx treatment carried out in real time on the Carrara marble are shown in Figure 1. The vibrational modes of the reagent are located at 1573, 1453, and 1307 cm^–1^ [spectrum (b) in Figure 1], and they are well distinguishable from those of liquid water [spectrum (a) in Figure 1] at ∼3700–3100 cm^–1^, at 1643 cm^–1^, and by the rising absorption band from 1000 to 400 cm^–1^. When a drop of AmOx solution is applied on the stone substrate [spectrum (c) in Figure 1], the absorption bands of calcite are detectable as well at 871 cm^–1^ ν_2_(CO_3_^2–^) and 709 cm^–1^ ν_4_(CO_3_^2–^). The ν_3_(CO_3_^2–^) marker band at ∼1391 cm^–1^ of calcite overlaps that of the AmOX solution at 1453 cm^–1^, and it can only be observed after the spectral subtraction with the ATR–FTIR spectrum of the AmOX solution [spectra (c,d) in Figure 1].^42^

The vibrational modes of newly formed calcium oxalates are visible in the ATR–FTIR spectra from the very first measurement. This suggests that the growth of calcium oxalate crystals starts as soon as the AmOX solution contacts the Carrara marble. Calcium oxalate nanocrystals display a different phase stability in the presence of “external” water molecules (e.g., under different hygrometric conditions), as shown by Conti et al,.^10^ Therefore, the vibrational modes of such reaction products were used to study the real-time kinetics of crystallization on the marble substrates directly in a water-based reaction environment.

The initial formation of weddellite [WD, CaC_2_O_4_·(2 + *x*)H_2_O] can be inferred by the spectral bands at 1322–1318 cm^–1^ [ν_as_(CO)], 778 cm^–1^ [δ_in-plane_(OCO)], 511 cm^–1^ [δ_in-plane_(OCO)], at 1615 cm^–1^ [ν_sy_(CO)], and the broad band at about 3469 cm^–1^ (OH stretching of structural water), just after the first measurement (Figure 1).^43^ WD is a metastable calcium oxalate. During the reaction, the bands of WD shifted at 1608, 1310, and 515 cm^–1^. This shift was the result of the transformation of WD to whewellite (WH, CaC_2_O_4_·H_2_O), the more stable monohydrated crystalline phase. The formation of WH from WD is also confirmed by the evolution of the OH stretching band. In fact, the broad band at 3469 cm^–1^ of WD (structural disorder of H_2_O molecules,^43^) evolves into the well-defined sequence of five bands at 3062, 3248, 3335, 3432, and 3482 cm^–1^ of WH (ordered hydrogen-bonded net^43^). WH crystals remained stable after the treatment, as demonstrated by the ATR–FTIR spectra collected after curing, washing, and drying of the stone substrate. These were novel findings with respect to the literature. In the paper by Conti et al., the phase transformation of WD to WH was detected at 273 K on the synthetic nanopowders of WD, therefore on a system having a very high specific surface and composed only by WD.^10^ Here, the WD-to-WH phase transformation was detected in the water solution and on the stone substrates. These conditions make the identification of the phases complex due to the copresence of calcite, liquid water, and AmOx, and due to the low-weight fraction of calcium oxalates with respect to the other compounds (calcite, water, and AmOx).

In addition, variations of calcite bands are detected after the treatments. In fact, the ν_3_(CO_3_^2–^) and ν_2_(CO_3_^2–^) modes are at 1391 and 871 cm^–1^, respectively, for the untreated calcite and at 1440–1408 and 864–860 cm^–1^, respectively, for treated calcite. This occurs for both the treatments. In the case of AmOx, the shift is more evident because the newly formed phases have no vibrational bands in these spectral regions and no carbonate substituents (Figure 1). Considering that the treatments dissolve submicrometric layers of calcite at the interface between the stone grains and the solutions, these peak shifts reflect a compositional—structural variation with the formation of a micrometer- or nanometer-size regions of amorphizated calcite.

**Figure 2:**
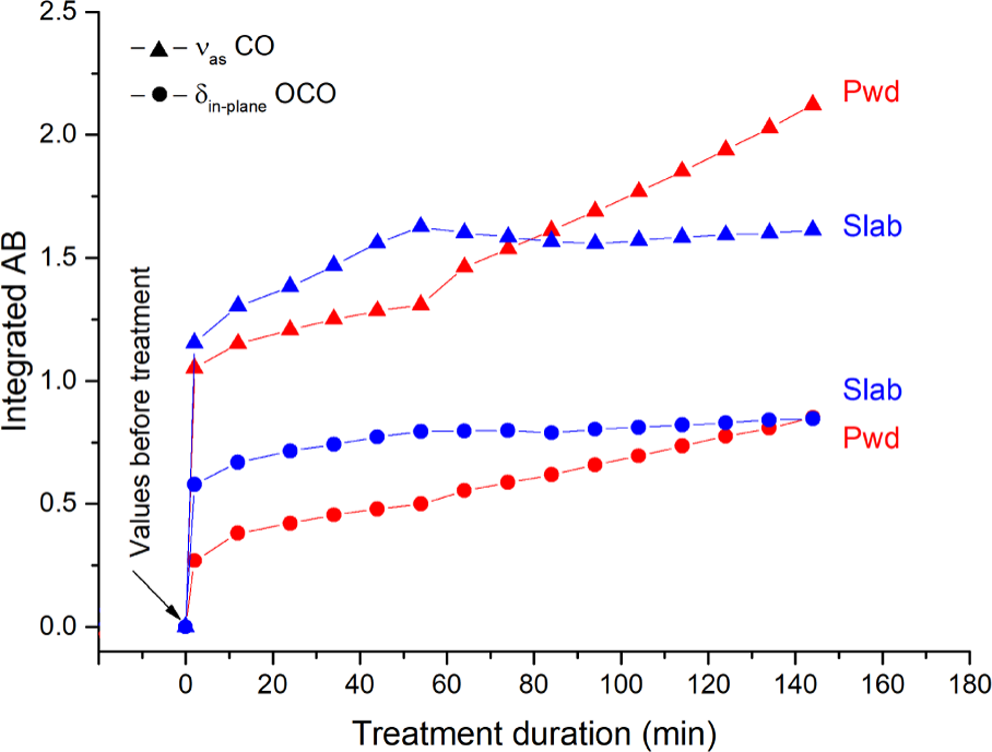
Integrated absorbance of the ν_as_(CO) (▲) and δ_in-plane_(OCO) (●) marker bands measured on powders (“Pwd”, red lines) and slab (“Slab”, blue lines) of the Carrara marble plotted versus the treatment duration. Integrated range for the ν_as_(CO) band: 1345.9–1247.5 cm^–1^. Integrated range for the δ_in-plane_(OCO) band: 810.05–725.19 cm^–1^.

WD and WH are formed on both slabs and powders, as shown in Figure S2. Some differences can be observed in the trend of crystallization, with powders giving rise to a more abundant crystallization than slabs due to the higher specific area (Figure S3). Furthermore, these differences are visualized in Figure 2, where the integrated absorbance of the δ_in-plane_(OCO) and ν_as_(CO) marker bands of calcium oxalates versus the treatment duration, as measured on the two substrates, are plotted.

The integrated absorbance of both the marker bands increases over time. This rise occurs for both stone substrates, and it indicates the progressive nucleation and growth of conservation products during the reaction. However, in the case of Carrara marble slab (blue lines in Figure 2), the rising trend of the integrated absorbance of marker bands continues up to 50–60 min from the beginning of the reaction. After that, the growth of this band reaches a plateau. This is probably due to the completion of the reaction, with the formation of a micrometer-size shell of calcium oxalates on the surface of all calcite grains exposed to the AmOx solution. This shell acts as a barrier and hinders the ion exchanges between the calcite grains and the surrounding solution, which is also the goal of the AmOx treatment. The shell is formed also on the powders but, on this substrate, the reaction does not reach a plateau and the integrated absorbance of marker bands for calcium oxalates increases with a linear trend during the whole ∼150 min of measurements. In addition, the crystallization of calcium oxalates on powders is so abundant that the characteristic bands of newly formed phases (1615–1608 and 1318–1310 cm^–1^) are well-resolved from that of the AmOx solution even without any spectral subtraction (Figure S3). This difference can be attributed to a higher crystallization rate occurring on powders originating from the higher specific area of calcite grains exposed to the treatment, which, in turn, releases a higher amount of free Ca^2+^ ions in the reaction environment than slabs. This means that, comparing treatments carried out with the same reagent and molarity, the reaction rate is higher for substrates having a higher specific surface area. These results demonstrate that the reactivity of substrates to inorganic treatments could be significantly affected by the cohesion and microstructure of the stone material. At the same time, ATR–FTIR spectroscopy can be used to investigate in real time the growth of the newly formed shell even on those substrates having a low specific surface exposed to the treatments and where the newly formed phases are minor phases with respect to the minerals of the stone substrate. This finding is important in the field of the application of analytical chemistry to the conservation of cultural heritage.

### Ammonium Phosphate (DAP) Treatments

**Figure 3:**
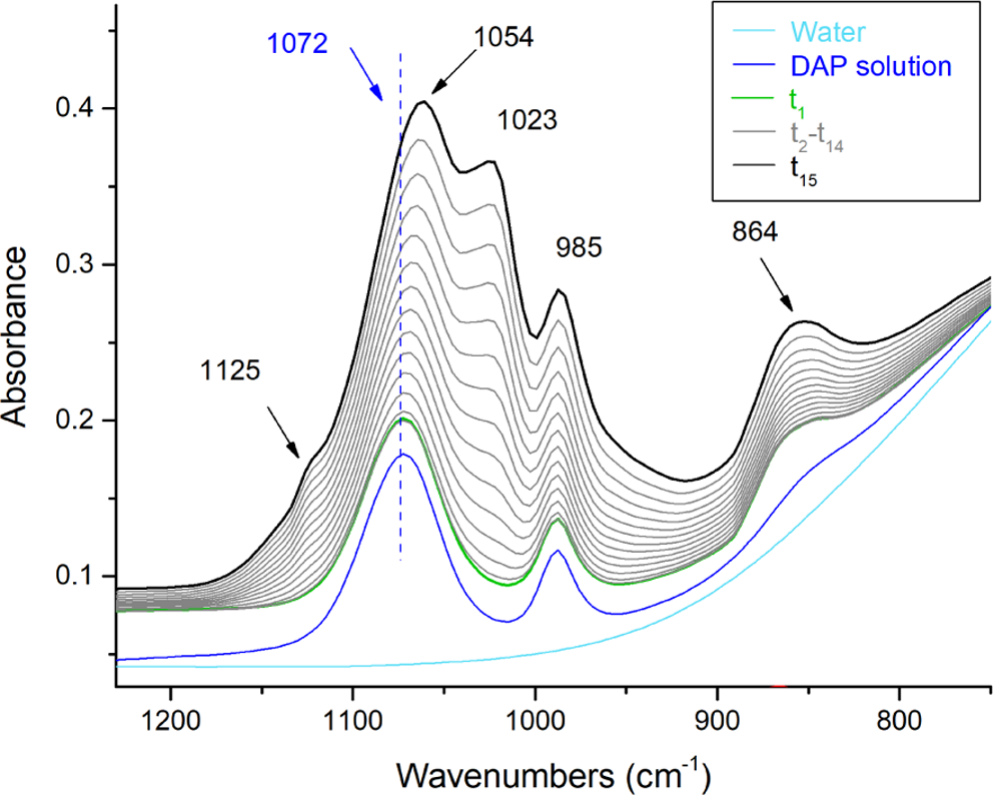
DAP treatment. ATR–FTIR spectra of water, of the DAP solution, and of the Carrara marble treated in real time at the beginning of the reaction (*t*_1_), during the reaction (*t*_2_–*t*_14_), and at the end of the reaction (*t*_15_).

In the DAP solution, the vibrational modes of the reagent are at 3245, 1455, 1072, 985, and 524 cm^–1^, and they are well distinguishable from those of water. When a drop of DAP solution is put on calcite of the Carrara marble substrate, new peaks are formed at 1125, 1023, 600, and 555 cm^–1^, and a peak shifts from 1072 cm^–1^ (due to the reagent) to 1054 cm^–1^. In addition, the increase of the absorbance of the peaks at 1451, 985, and 864 cm^–1^ was also observed. These variations are attributed to the formation of calcium phosphates, and they are used to study the crystallization of newly formed reaction products in real time (Figure 3).

A mixture of different crystalline phases can be identified by the ATR–FTIR spectra. In particular, the peaks at 1023 cm^–1^ [ν_3_(PO_4_^3–^)] and 600 and 555 cm^–1^ [both ν_4_(PO_4_^3–^)] in Figures 3 and S4 are due to the crystal growth of hydroxyapatite (HAP, Ca_10_(PO_4_)_6_(OH)_2_).^42,44^

Apatites have a high-compositional flexibility, which can accommodate a large number of ionic substituents, both cations and anions, and ionic vacancies. A particular type of anion substitution involves carbonate groups,^8,28^ which may be embedded in A sites (hydroxyl channel), B sites (phosphate site), or both AB crystallographic sites to form A-type, B-type, or AB-type carbonate HAP (C-HAP).^45,46^

The formation of C-HAP after DAP treatments on the Carrara marble is highly likely, as hypothesized (but not yet demonstrated) in a previous study based on synchrotron radiation X-ray diffraction.^3^ In fact, distinguishing a stoichiometric HAP from a nonstoichiometric C-HAP is quite challenging even using XRD,^44,47,48^ especially when this distinction needs to be carried out in the presence of calcite. It is crucial to define if the DAP treatment forms only HAP or a mixture of HAP and C-HAP. In fact, C-HAP is a metastable phase and it tends to transform into HAP.^2^ This transformation takes time and requires specific reaction conditions. It means that there will be a “curing” time, during which the composition of reaction phases is in evolution toward more stable crystalline phases even after the DAP treatment.

**Figure 4:**
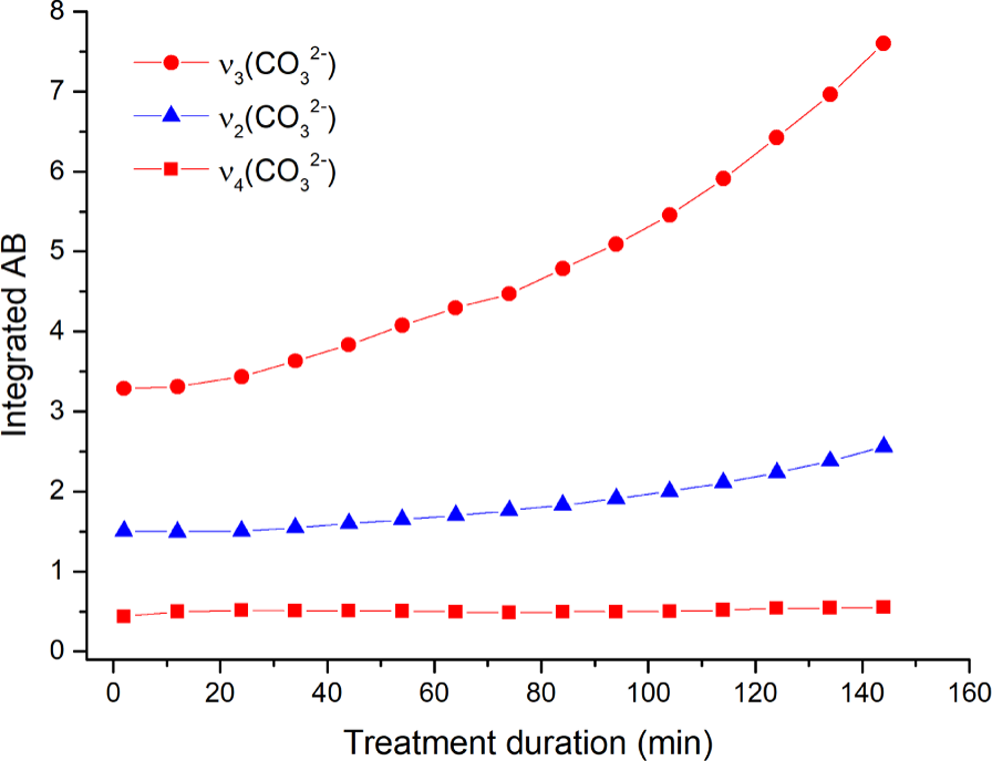
Integrated absorbance of the ν_3_(CO_3_^2–^), ν_2_(CO_3_^2–^), and ν_4_(CO_3_^2–^) vibrational bands measured during the DAP treatment on the surface of the Carrara marble slab plotted versus the treatment duration. Integrated ranges are as follows: 1519.0–1336.1 cm^–1^ for the ν_3_(CO_3_^2–^); 891.1–783.87 cm^–1^ for the ν_2_(CO_3_^2–^); and 724.08–686.53 cm^–1^ for the ν_4_(CO_3_^2–^).

Here, the formation of a nonstoichiometric poorly crystalline C-HAP is finally inferred, thanks to the high chemical selectivity of ATR-FTIR spectroscopy and despite the copresence of calcite. This identification is based on the trend of the absorbance of carbonate modes in the ATR–FTIR spectra during the reaction (Figure 4).

In detail, the marker bands of calcite are at 1394 cm^–1^ ν_3_(CO_3_^2–^), 871 cm^–1^ ν_2_(CO_3_^2–^), and 709 cm^–1^ ν_4_(CO_3_^2–^). C-HAPs have peculiar ν_3_(CO_3_^2–^) and ν_2_(CO_3_^2–^) broad vibrational modes between ∼1460–1400 and ∼880–870 cm^–1^(depending on the A-, B-, or AB-type substitution^42,49^), respectively, and they are therefore overlapped with calcite ones. Only the peak at 709 cm^–1^ due to the ν_4_(CO_3_^2–^) is unique to calcite.^42^ The absorbance of ν_4_(CO_3_^2–^) remains constant during the whole reaction (Figure 4), indicating that the surface of calcite crystals is partially dissolved by the reagent (calcite is the source of calcium ions for the crystallization of calcium phosphates), but this dissolution does not dramatically affect the relative amount of calcite present. Otherwise, a dramatic decrease of the amount of calcite should significantly decrease the absorbance of the ν_4_(CO_3_^2–^) vibrational mode over time.

As for the other (CO_3_^2–^) vibrational modes, a visible increase in the absorbance of the spectral bands of ν_3_(CO_3_^2–^) and ν_2_(CO_3_^2–^) is observed during the reaction, and this increase occurs in parallel to the increase of the ν_3_(PO_4_^3–^) band of apatite at 1023 cm^–1^ (Figures 3 and S4). This demonstrates that, even considering that a partial contribution of calcite to the absorbance of ν_3_(CO_3_^2–^) and ν_2_(CO_3_^2–^) bands cannot be excluded, the newly formed apatite is a carbonate-substituted HAP. Furthermore, considering the position of carbonate bands at ∼873 cm^–1^ ν_2_(CO_3_^2–^)^50^ and ∼1451–1415 cm^–1^ ν_3_(CO_3_^2–^),^42^ it can be hypothesized that the main formation is of B-type C-HAP.^28,42,49^

The formation of C-HAP is also confirmed by the absence of the OH^–^ stretching and libration modes at 3570 and 630 cm^–1^, typical of structural hydroxyl groups of stoichiometric crystalline HAP,^44,48^ in all the ATR–FTIR spectra. These findings support the literature data, in which the formation of a mixture of stoichiometric HAP (demonstrated) and nonstoichiometric C-HAP (hypothesized) from the DAP treatments was proposed.^3,9^ Herein, the mixture of stoichiometric and nonstoichiometric apatites will be referred in the following as HAP + C-HAP.

Dicalcium phosphate dihydrate (DCPD, CaHPO_4_·2H_2_O) crystallizes simultaneously to HAP + C-HAP as pointed out by the bands at 1125 cm^–1^ ν_3_(HPO_4_^2–^), 1061 cm^–1^ ν_3_(PO_4_), and 985 cm^–1^ ν_1_(PO_4_) (Figure S4). DCPD crystals grow during the whole DAP reaction as shown by the vibrational bands in Figures 3 and S4 (spectrum *t*_15_).^44^ The presence of DCPD is meaningful, as it provides information on the reaction condition. In fact, DCPD is a metastable crystalline phase, it has a calcium–phosphorous (Ca/P) molar ratio of 1 and is generally formed in acidic solutions with a low availability of free Ca^2+^ ions.^2,8^ In contrast, apatites have a higher Ca/P molar ratio (1.5–1.67 depending on the stoichiometry) and they are preferentially formed in neutral or basic conditions.^2,8^ Therefore, the copresence of DCPD with apatites and the formation of apatite in a nonstoichiometric C-HAP form demonstrate that these phases nucleate in not ideal reaction conditions and that they mutually compete for free Ca^2+^ ions.^3,9^

DCPD crystals have occasionally been detected on carbonatic stone substrates after DAP treatments in particular conditions: for example, on dolostones^6^ and magnesium-containing veins,^51^ after DAP treatments with prolonged duration.^3,9^ However, DCPD crystals were no longer found on treated stone substrates after a few months.

Here, the formation of DCPD is demonstrated for the first time during the DAP reaction and in its early stages in real time. Considering its metastability, the possible effects of additional water molecules has been checked as well. The ATR–FTIR spectroscopic investigations show that the bands of DCPD are no more present after the post-treatment washing and drying, and only the vibrational features of HAP + C-HAP can be observed (Figure S4, pattern “Dry”). These findings reveal the transformation of DCPD in apatite, and they support the literature data where DCPD is implicated as a possible precursor in the formation of more stable phases, like apatites.^2^

It is therefore reasonable to hypothesize the following: (i) DCPD always forms alongside HAP + C-HAP during DAP treatments, promoting the formation of poorly crystalline nonstoichiometric apatites and (ii) the washing of treated substrates is a crucial step to promote the formation of more stable and insoluble phases (HAP + C-HAP).

The investigation of the distribution of newly formed phases during the DAP reaction is as important as their crystallo-chemical characterization.

**Figure 5:**
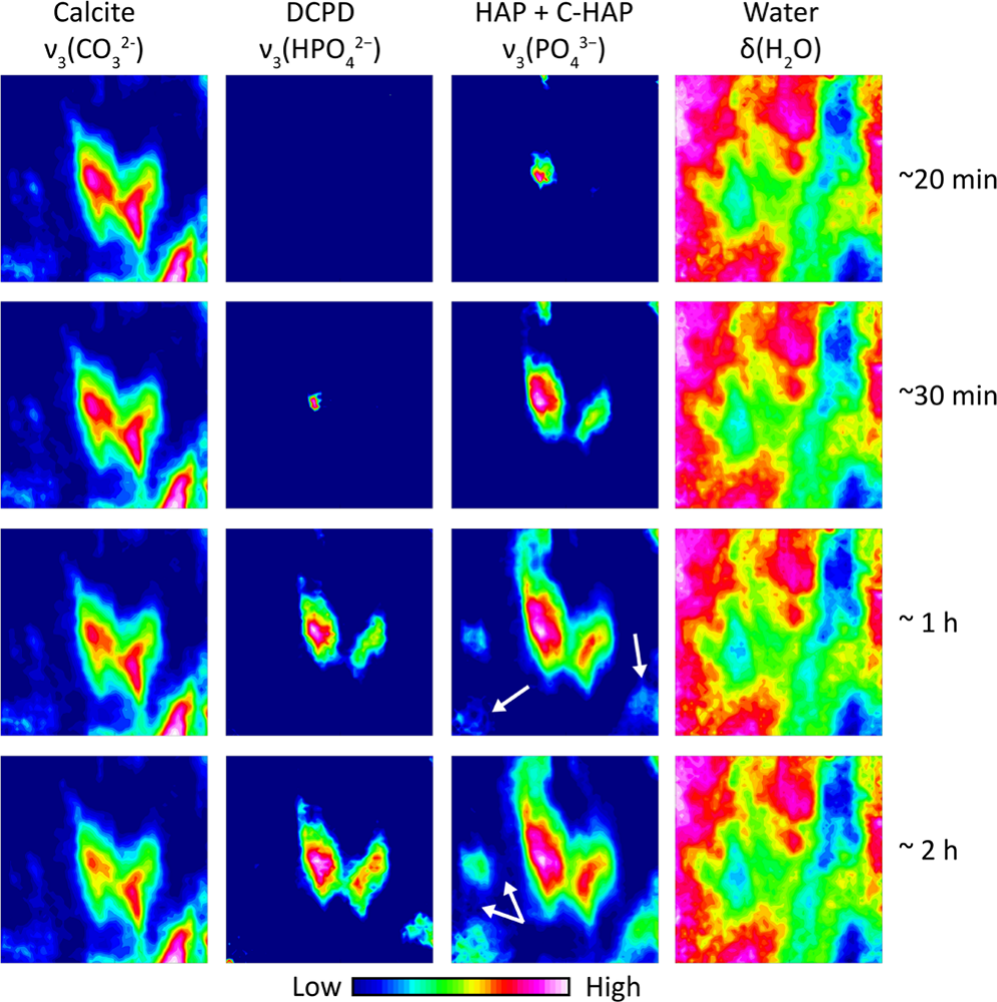
Macro ATR–FTIR spectroscopic images of Carrara marble powders treated by DAP in real time. The chemical images show the spatial distribution of the ν_3_(CO_3_^2–^) of calcite (1400 cm^–1^), ν_3_(HPO_4_^2–^) of DCPD (1125 cm^–1^), ν_3_(PO_4_^3–^) of HAP and C-HAP (1023 cm^–1^), and ν_3_ (H_2_O) of the water-based DAP solution (1636 cm^–1^) in relation to the different treatment time (∼20 min, ∼30 min, ∼1 h, and ∼2 h). The white arrows in HAP chemical images indicate the clear formation of the apatite crystal network after ∼1 h of the DAP treatment. The imaging area is ∼0.6 mm (vertical) × 0.55 mm (horizontal).

Figure 5 shows the spatial distribution of calcite, HAP + C-HAP, and DCPD during the DAP reaction. As FPA detectors have the typical low-wavenumber spectral region cutoff below 900 cm^–1^, it was not possible to consider some marker bands in the fingerprint region. Therefore, the spatial distribution of calcite, HAP + C-HAP, and DCPD was inferred by integrating their ν_3_(CO_3_^2–^), ν_3_(PO_4_^3–^), and ν_3_(HPO_4_^2–^) vibrational modes, respectively.

The initial morphology and spatial distribution of calcite grains is shown in the chemical images (Figure 5). Calcite grains are well surrounded by the aqueous DAP solution during the whole reaction. The shape of stone grains remains unvaried during the whole reaction, which indicates the very minor action of dissolution carried out by the reagent. Nevertheless, this minor dissolution is sufficient to form HAP + C-HAP and DCPD crystals, as previously discussed, and they can be detected in the chemical images of Figure 5 after ∼20 and ∼30 min from the beginning of the treatment. The crystallization time deduced by the chemical images appears with a slight delay with respect to conventional ATR–FTIR spectra. However, it is worth noting that these chemical images are obtained by integrating the marker bands without the spectral subtraction of the DAP solution. This implies that the vibrational bands of the DAP solution partially overlapped with those of newly formed phases. In fact, the crystallization needs to be more pronounced to be clearly detected in chemical images, which explains this ostensible delay in the crystallization.

Focusing on the localization of calcium phosphates, DCPD crystals grow from calcite grains and remained quite confined/overlapped with them. Similarly, HAP + C-HAP phases nucleate at early stages on the surface of calcite grains. However, as long as the reaction continues, HAP + C-HAP crystals develop themselves in the 3D space, by covering the whole surface of calcite grains (the so-called shell)^3,9^ and by creating a framework of apatite in the voids of the stone substrate with the typical rose-like/bone-like morphology of the HAP + C-HAP crystal network (see the white arrows in Figure 5).^3,9^ DCPD does not contribute to the formation of the consolidation framework in a treatment having the duration investigated in this study. As a result, HAP + C-HAP would seem to be the main crystalline phases to create the consolidation framework.

## Conclusions

This study demonstrated the feasibility, practicality, and potential of advanced ATR–FTIR spectroscopy and spectroscopic imaging approaches to investigate an unexplored topic in conservation science: the time-resolved, chemical, and spatial investigation of the reaction of inorganic conservation treatments (AmOx and DAP) with carbonatic substrates directly in water-based solutions.

The new analytical data obtained by ATR–FTIR spectroscopy with a single element detector and macro ATR–FTIR spectroscopic imaging with an array detector complemented each other. This ATR–FTIR spectroscopic approach allowed the composition, spatial distribution, kinetics of crystallization, and the phase variation of reaction products, to be identified since the very early stages of the reaction. For the first time, the nucleation and spatial development of the crystal network, providing the consolidating action (shell), has been shown through a time-resolved sequence of chemical images. The crystallization of stable and metastable reaction products, the transformations induced by the treatments to the stone substrate, and the effects induced by post-treatment washing have been critically discussed. The new analytical information obtained in this study increases our knowledge on conservation treatments, permitting us to develop more effective conservation procedures.

By showing this new analytical application of ATR–FTIR spectroscopic techniques, our research overcame a demanding analytical challenge in conservation science: the characterization and localization of conservation products having a very similar chemical composition, in complex mixture, during their crystallization and directly in an aqueous reaction environment. Therefore, this research points out the novel possibility to apply our ATR–FTIR spectroscopic approach, and of macro ATR–FTIR spectroscopic imaging in particular, to study by chemical images, the reactions taking place during conservation treatments on cultural heritage substrates. Moreover, the presence of liquid water (or a liquid in general, even if IR-active) between the stone substrate and the ATR crystal significantly helps to minimize the possible issues with contact in the measurement of irregular and hard surfaces (typically common for the stone materials of cultural heritage). These outstanding results can be achieved avoiding complex and destructive sample preparations and without resorting to advanced XRD techniques with synchrotron radiation.^3,7,9,51^

Above all, the strength of this study is that it gives rise to novel analytical perspectives. In fact, by highlighting the remarkable advantages of using both ATR–FTIR spectroscopy and spectroscopic imaging to study systems that change during a chemical reaction and directly in the solvent-based reaction environment, our study provides new analytical tools to address the choices of conservation treatments in pilot worksites and opens new analytical scenarios in conservation science and analytical chemistry.

## Supporting Information Available

Experimental setup and time line of the experiments and ATR–FTIR spectra showing the crystallization of calcium oxalates and calcium phosphates during the AmOx and DAP treatments (PDF)
