# Supplementary material for: Time-Resolved ATR–FTIR Spectroscopy and Macro ATR–FTIR Spectroscopic Imaging of Inorganic Treatments for Stone Conservation
Source: Anal Chem. 2021 Oct 26;93(44):14635–42. doi: 10.1021/acs.analchem.1c02392 (PMC9295121; doi:10.1021/acs.analchem.1c02392)
Supplement: Supplementary file 1 — ac1c02392_si_001.pdf [file ac1c02392_si_001.pdf]

Supporting Information

Time-Resolved ATR-FTIR Spectroscopy and Macro ATR-FTIR Spectroscopic Imaging of Inorganic Treatments for Stone Conservation

Elena Possenti[1,*], Chiara Colombo[1], Marco Realini[1], Cai Li Song[2,‡] and Sergei G. Kazarian[2,*]

[1]*Istituto di Scienze del Patrimonio Culturale, Consiglio Nazionale delle Ricerche, ISPC-CNR, Via R. Cozzi 53 Milano, 20125, Italy*

[2] *Imperial College London, Department of Chemical Engineering, South Kensington Campus London, SW7 2AZ, United Kingdom*

* Corresponding authors: elena.possenti@cnr.it

s.kazarian@imperial.ac.uk

‡ Current address: PETRONAS Research Sdn Bhd, Jalan Ayer Itam, Kawasan Institusi Bangi, 43000, Bandar Baru Bangi, Selangor, Malaysia

Table of Contents

Figure SM1 Experimental setup and time line of the experiments carried out in real time by conventional ATR-FTIR spectroscopy and macro ATR-FTIR spectroscopic imaging.

Page S3

Figure SM2 ATR FTIR spectra showing the crystallization of weddellite (black lines) and whewellite (red lines) both on Carrara marble slabs ("Slab", dotted lines) as well as on Carrara marble powders ("Pwd", solid lines). "t_1" and "t_{15}" refer to ATR-FTIR spectra collected in real time after 2'30'' and 142'30'' from the beginning of the treatment.

Page S4

Figure SM3 ATR-FTIR spectra collected in real time during the AmOx treatment showing the growing absorbance of bands (1608 cm^{-1}, 1310 cm^{-1}) due to calcium oxalates formed on Carrara marble slab (on the left) and powders (on the right).

Page S5

Figure SM4 ATR FTIR spectra showing the crystallization of calcium phosphates on Carrara marble during the last measurement of the treatment (t_{15}, after 142'30'' of reaction) and after the post-treatment washing and drying (Dry). The ATR-FTIR spectrum t_{15} is obtained with the spectral subtraction of the DAP solution.

Page S6

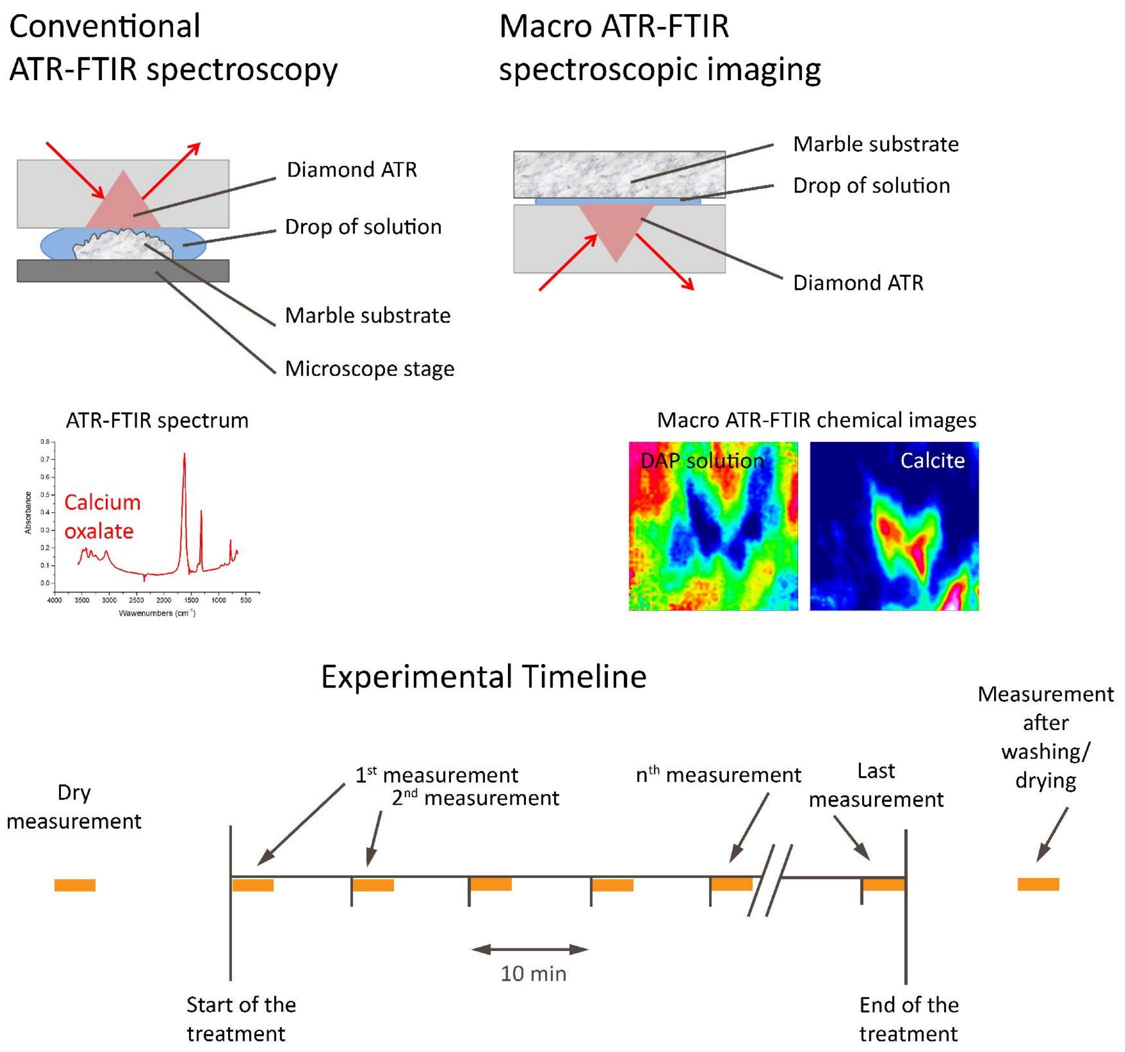

Figure SM1. Experimental setup and time line of the experiments carried out in real time by conventional ATR-FTIR spectroscopy and macro ATR-FTIR spectroscopic imaging.

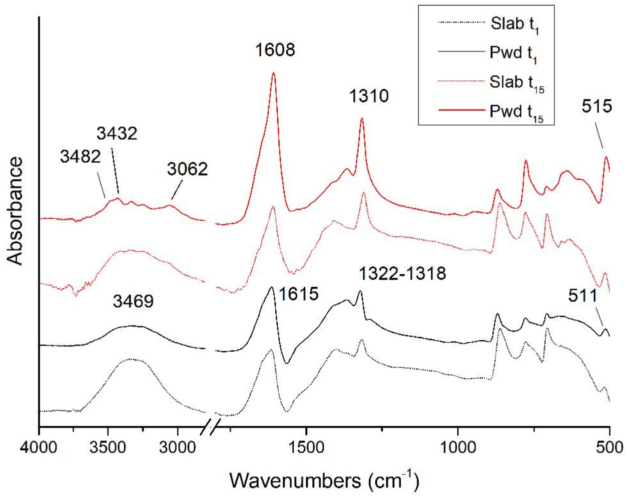

Figure SM2. ATR FTIR spectra showing the crystallization of weddellite (black lines) and whewellite (red lines) both on Carrara marble slabs (“Slab”, dotted lines) as well as on Carrara marble powders (“Pwd”, solid lines). “t_1” and “t_{15}” refer to ATR-FTIR spectra collected in real time after 2’30’’ and 142’30’’ from the beginning of the treatment.

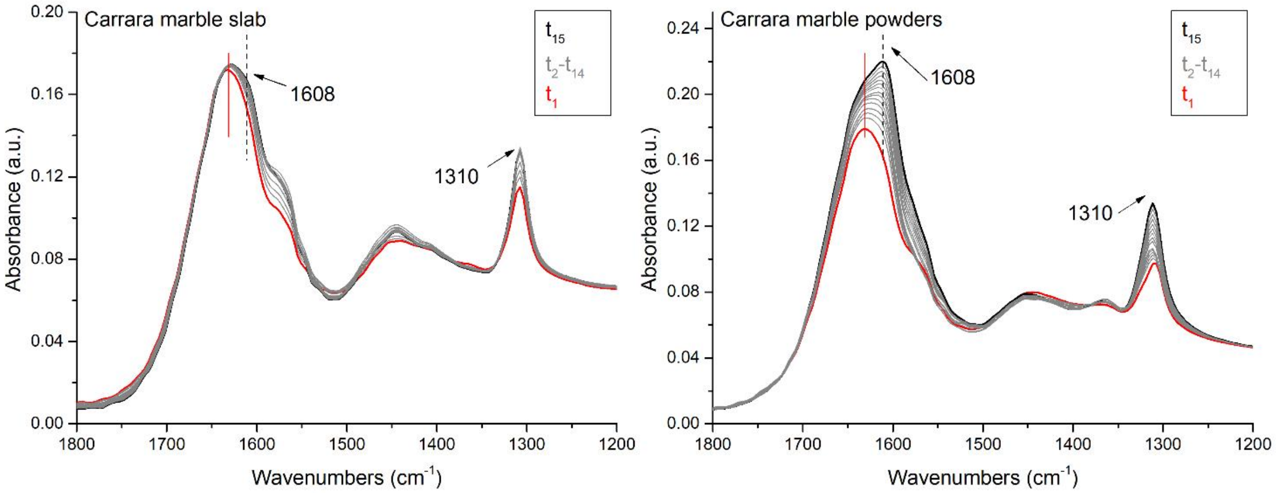

Figure SM3. ATR-FTIR spectra collected in real time during the AmOx treatment showing the growing absorbance of bands (1608 cm^{-1}, 1310 cm^{-1}) due to calcium oxalates formed on Carrara marble slab (on the left) and powders (on the right).

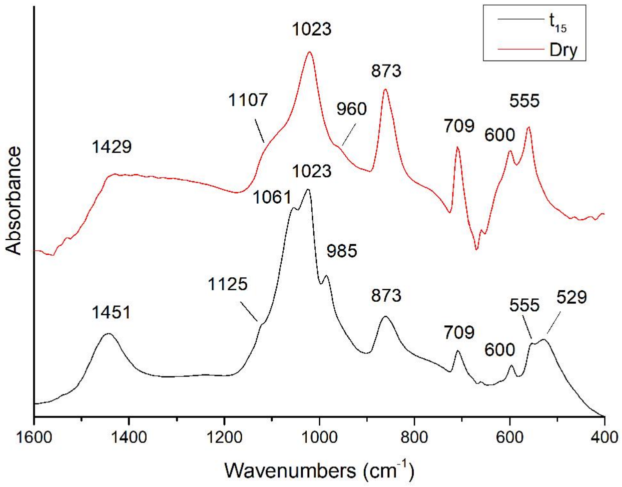

Figure SM4. ATR FTIR spectra showing the crystallization of calcium phosphates on Carrara marble during the last measurement of the treatment (t_{15}, after 142’30’’ of reaction) and after the post-treatment washing and drying (Dry). The ATR-FTIR spectrum t_{15} is obtained with the spectral subtraction of the DAP solution.
